# Towards the standardization of human platelet lysate production and its comparison to fetal bovine serum for human hematopoietic cell culture: a scoping review

**DOI:** 10.3389/ftox.2025.1496231

**Published:** 2025-06-27

**Authors:** Shamili Immalaraju, Srishti Goyal, Rukmini Jonnalagadda

**Affiliations:** Cell Culture R&D, ULQA Scientific, Bangalore, India

**Keywords:** fetal bovine serum (FBS), human platelet lysate (HPL), human hematopoietic cells, fold expansion, cell culture stability, xeno-free (XF)

## Abstract

Human hematopoietic cell culture (HCC) refers to the *ex vivo* growth of normal cells of the hematological system. These cells can be used as models to understand hematopoiesis and related malignancies. HCC also holds immense potential to help develop safer vaccines and immunotherapies, as well as donor-independent blood products. *In vivo*, these cells grow and differentiate in highly specialized conditions but replicating these *in vitro* is a significant technical challenge. Although various strategies have been developed to optimize HCC expansion, implementing them can be costly. Consequently, traditional fetal bovine serum (FBS)-containing media is the first choice, despite its disadvantages. Over the past two decades, human platelet lysate (hPL) has emerged as a viable alternative. However, variations in protocols and reporting standards across laboratories have resulted in a mixed picture regarding its feasibility to replace FBS. Thus, this study aimed to review existing literature that directly compared HCC performance in hPL and FBS supplementation. PubMed, Google Scholar, and the FCS-free database were queried between 1 January to 30 July 2024. Using pre-defined inclusion and exclusion criteria, five out of 622 relevant records were included in this scoping review. Data on the hPL production method, HCC conditions and performance were extracted. We identified gaps in the consideration of key hPL production parameters and recommend addressing them to reduce the variability observed in hPL performance. Even though hPL production parameters were repeatedly overlooked, hPL outperformed FBS supplementation in terms of cell identity and functionality across the included HCC studies. Therefore, we highlight the potential of these recommendations to overcome existing technical challenges in HCC, as well as support the development of effective FBS alternatives by enhancing the reproducibility and reporting standards of future studies.

## 1 Introduction

Hematopoietic cell culture (HCC) refers to the *ex vivo* growth and expansion of normal cells that constitute the hematological system (Bozhilov et al., 2023). *In vivo*, these cells exist in complex microenvironments and are maintained through highly regulated pathways (Weijts et al., 2021). The dysregulation of these conditions can lead to hematological disorders (Hu and Ali, 2016; Ribeiro-Filho et al., 2019). Several strategies have been applied to grow and differentiate normal hematopoietic cells to obtain significant volumes. These strategies have been reviewed elsewhere and can range from complex bioreactors to the addition of small molecules in simpler culture systems (Rubio-Lara et al., 2023; Branco et al., 2024). Nevertheless, advances in HCC are essential to enhancing our fundamental knowledge of human hematopoiesis (Gupta et al., 2024). HCC also holds immense potential to improve the safety and efficacy of existing bone marrow transplantation, immunotherapies, gene therapies, and the production of blood products (Epah and Schäfer, 2021; Hassan and Rajput, 2024).

There is a growing emphasis on developing serum-free media (SFM) to produce functional hematopoietic cells *ex vivo*. When comparing SFM to traditional media with fetal bovine serum (FBS), their performance is highly context dependent (Choi et al., 2010; Jeon et al., 2010; Sutton et al., 2016). SFM (UltraCulture™, Lonza) was effective for expanding umbilical cord blood (UCB)-derived CD34^+^ cells, especially in terms of total cell numbers; however, IMDM, with or without FBS, was superior in supporting the expansion of functionally potent colony forming unit (CFU), especially CFU-Granulocyte Macrophage (Choi et al., 2010). Another study showed that the fold expansion of γδ T cells cultured from peripheral blood mononuclear cells (PBMCs) in SFM (OpTmizer™, Thermo Fisher Scientific) was approximately 30%–35% of that observed in the RPMI-1640 with 10% FBS (Sutton et al., 2016). However, cells grown in SFM dramatically upregulated low-density lipoprotein receptors and were a better candidate for transduction compared to cells supplemented with FBS. Meanwhile, Jeon et al. (2010) used statistical modeling to optimize SFM (AIM-V, Invitrogen), through cholesterol and polyamine addition, resulting in a 1.5-fold higher yield of viable cytotoxic T lymphocytes (CTLs) from PBMCs compared to RPMI-1640 with 10% FBS. Together, these studies highlight that while SFM offers safe and consistent media, it is still a minimal media that may not be suitable for applications where yield, stemness, and multilineage potential are a priority.

A recent review revealed that only a quarter of the published HCC studies used SFM (Yadav et al., 2020). In most cases, the formulation details of commercially available SFM are proprietary and cannot be considered fully defined (van der Valk et al., 2010). As SFM do not offer the same level of complexity as traditional supplements and formulation details that remain unknown to the researcher, it is easy to overlook cellular penalties and stress caused by SFM. Kruta et al. (2021) demonstrated that SFM (StemSpan™ SFEM) induced proteostatic stress, which can compromise cell function and physiological relevance. In addition to the high cost associated with SFM, the need to further optimize them to obtain clinically relevant cell volumes is a significant hurdle for the widespread acceptance of SFM (Bastani et al., 2023). Therefore, FBS-supplemented media is still the preferred option for many researchers (Yadav et al., 2020; Cassotta et al., 2022). The use of FBS is associated with serious implications regarding its ethical sourcing (Jochems et al., 2002), transparency in its supply chain (Gstraunthaler et al., 2013), and reproducibility of resulting cell culture experiments (Baker, 2016). In addition to these concerns, there is a strong possibility that the industry has reached “peak serum”— a point where the FBS supply can no longer match the rapidly increasing demand (Brindley et al., 2012). Further, increasing yield to clinically relevant volumes require substantial resources and optimization, which limits widespread adoption of existing SFM platforms.

Over the years, human-derived blood products, or xeno-free supplements, namely, human platelet lysate (hPL), have become an attractive option to replace FBS in many human cell culture applications (Rauch et al., 2011; van der Valk, 2018; Rojas et al., 2024). Doucet et al. (2005) first proposed using hPL as an FBS alternative in human mesenchymal stem cells (MSC). Rauch et al. (2011) demonstrated that hPL prepared from expired donations effectively supported the growth and maintenance of various immortal cell lines. The culture performance was comparable to or even surpassed that of FBS in some cases. Additionally, the activation of the ERK1/2 MAPK pathways in cultured cells was similar to that observed with FBS supplementation. Several studies have since emerged showing improved cell proliferation behavior, an increased proportion of cells expressing characteristic immunophenotypes, and increased differentiation potential compared to FBS supplementation (Burnouf et al., 2016; Guiotto et al., 2020). It is also an effective replacement in other cell types including endothelial colony-forming progenitors and induced pluripotent stem cells (Barro et al., 2020; Peters et al., 2022), which suggests its potential to replace FBS in various other cell culture applications. Therefore, hPL may offer an effective solution to overcome challenges regarding the expansion and scaling of HCC but an evaluation of existing literature is missing. This review aims to evaluate the current state of HCC, focusing on the efficacy and performance of hPL compared to FBS supplementation. It examines the progress made, while also discussing the ongoing challenges and future directions for improving hPL supplementation in HCC.

## 2 Methods

### 2.1 Protocol

This study was conducted following the methodological guidance provided by the Joanna Briggs Institute for scoping reviews and is reported in accordance with the PRISMA-ScR (Preferred Reporting Items for Systematic Reviews and Meta-Analyses extension for Scoping Reviews) checklist, which promotes transparency and rigor in scoping review reporting (Peters et al., 2020; Tricco et al., 2018).

### 2.2 Eligibility criteria

Eligibility criteria were established as *a priori* to include original research articles in the review. Articles were screened by three investigators based on their titles and abstracts. Next, the full texts of all the records that contained terms related to human HCC were collected and uploaded into Zotero version 7.0.3 (https://www.zotero.org/). The methodology of each was independently reviewed by two investigators. Articles that were included (1) directly compared hPL with FBS in HCC (2) involved culture in plastic-only (e.g., not coated/treated), containers, and (3) were written in English. Articles were excluded if they (1) did not include an FBS control, (2) declared previous contamination with FBS before culture in the alternative, and (3) used a different basal media for the alternative and FBS. Discrepancies between the findings of the two investigators were resolved by consulting with the third investigator.

### 2.3 Information sources

A structured search was performed, by querying three electronic databases: PubMed, Google Scholar, and the FCS-free database from 1 January to 30 July 2024. The relevant MeSH terms and entry terms were based on the PICO (population, intervention, comparison, and outcome)-structured question: “Could human platelet lysate be applied as an FBS substitute?”, where, P: human HCC; I: human platelet lysate (hPL); C: FBS; and O: HCC performance characteristics, including fold expansion, viability, identity, and functionality.

### 2.4 Search

To query PubMed and Google Scholar, using the search syntax, “(Hematopoietic Stem Cells OR Hematopoietic Cells) AND (Cell Culture Techniques OR Cell Cultures) AND (Fetal Bovine Serum OR FBS OR Fetal Calf Serum OR FCS) AND (Serum-Free Media OR Xeno-Free Media OR Xeno-Free Supplement) AND (Human Platelet Lysate OR hPL OR Platelet Lysate OR Human Serum OR AB Serum OR Pooled Plasma) AND (Cell Proliferation OR Cell Expansion OR Cell Viability)” resulted in several hits including other cell types. Consequently, the search was broadened by removing “(Hematopoietic Stem Cells OR Hematopoietic Cells) AND.” To retrieve articles from the FCS-free database (https://fcs-free.sites.uu.nl/), the “literature” and “mammalian” filters were selected under source and species, respectively. A collection of records was compiled using Microsoft Excel and duplicate data was not included.

### 2.5 Data charting process

Two investigators performed data extraction independently. For the included studies, relevant data were extracted from the main text, tables, and figures, as well as supplementary files. The following data were collected for each study: (1) hPL production method; (2) supplement concentration; (3) cell morphology; (4) cell viability; (5) fold expansion data; (6) cell identity; and (7) functional assays.

### 2.6 Data items

Variables in the hPL production protocols were identified across studies. The cytokine regimes, basal media, and other additives were also collected, along with the concentration of hPL or FBS supplementation. The following parameters were qualitatively analyzed across the included studies: fold expansion, cell viability, identity evaluation, and functional assays.

## 3 Results

### 3.1 Selection of sources of evidence

**FIGURE 1:**
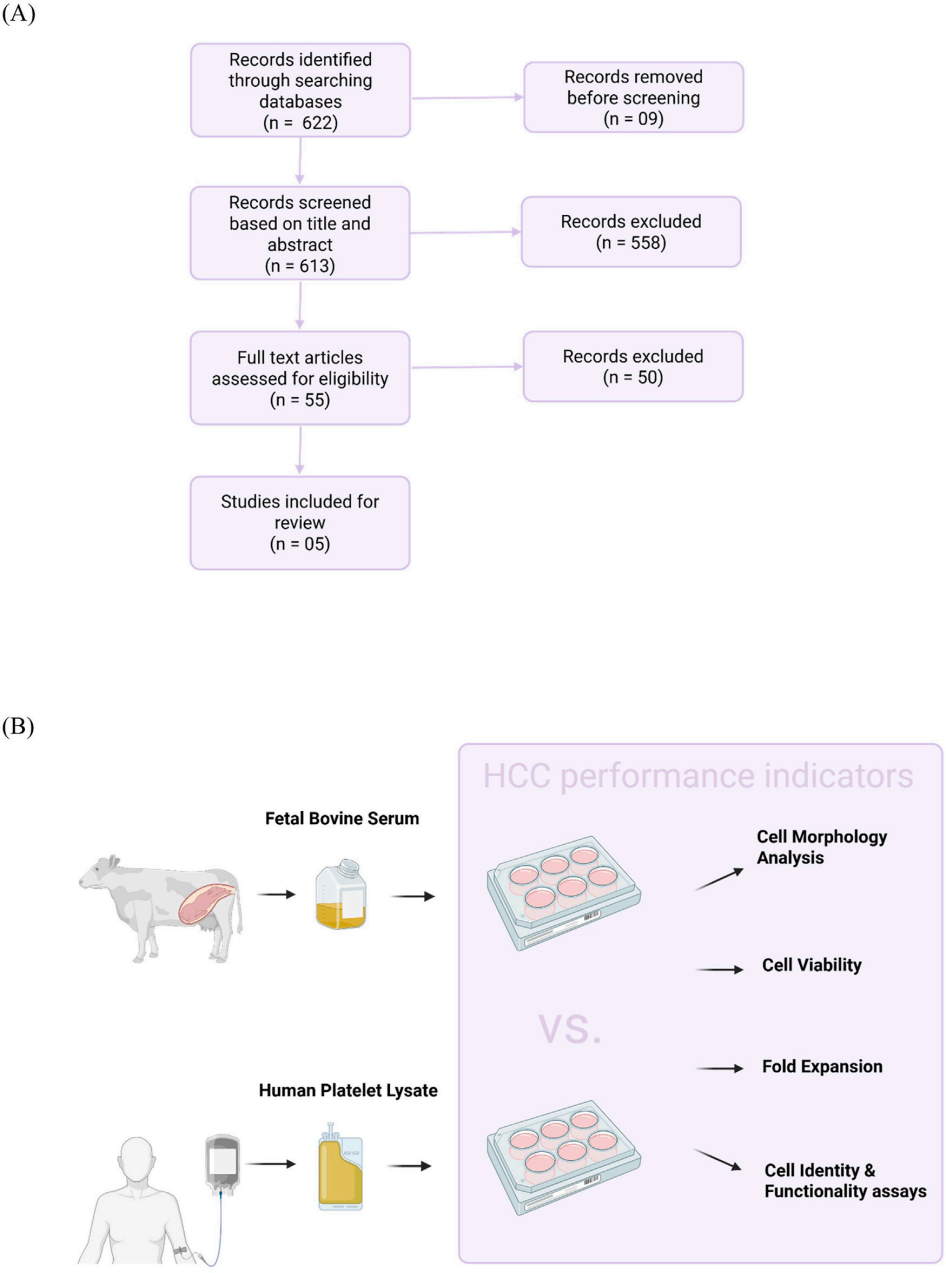
Selection of sources of evidence. **(A)** Flow diagram illustrates the selection of studies for this scoping review. PubMed, Google Scholar, and the FCS-free database were queried in the period between 1 January and 30 July 2024. Using pre-defined inclusion and exclusion criteria, five out of 622 relevant records were included. **(B)** Experimental design of included studies. Data on human platelet lysate production methodology and human hematopoietic cell culture (HCC) conditions and performance indicators were collected from the included studies.

As shown in Figure 1A, the initial search identified 622 relevant records. Nine records were removed before screening, including duplicates and non-English language texts. Another 558 records were excluded based on screening their titles and abstracts. The full texts of 55 records were analyzed and 50 did not satisfy one or more pre-defined criteria. Finally, five studies that compared hPL and FBS groups were included in this review. Data were extracted from two main areas: hPL production method as well as HCC conditions and performance indicators (Figure 1B).

### 3.2 Cell morphology

As it provides information on cell health and behavior, cell morphology is an important phenotypic indicator of HCC performance. However, only two studies compared the effects of hPL and FBS supplementation on cell morphology using inverted light microscopy (Švajger, 2017; Jabbarpour et al., 2023). Švajger (2017) examined the morphology of dendritic cells (DCs) after differentiation from peripheral blood mononuclear cells (PBMCs; Day 6) and activation into other subtypes (Day 8). On Day 6, circular monocytes became ‘potato-like’ during their differentiation to immature DCs (iDCs) in both FBS and hPL supplemented cultures, with no observable morphological differences. Additionally, no morphological differences were observed after the activation of iDCs to mature dendritic cells (mDCs) and tolerogenic dendritic cells (TolDCS) using lipopolysaccharide and 1,25-dihydroxyvitamin D3, respectively. Thus, this study reported equal efficacy in maintaining the morphology of DC subtypes using FBS and hPL at 10% supplementation.

Meanwhile, Jabbarpour et al. (2023) reported observable morphological differences between FBS and hPL supplemented cytokine-induced killer (CIK) cells, after differentiation from PBMCs (Day 15). Three supplement concentrations were tested (2.5, 5, and 10%). Compared to 10%, fewer aggregates were observed at lower concentrations in both FBS and hPL, supporting that supplement concentration directly affects cell proliferation. However, aggregates were notably denser and irregular in size and shape across all three concentrations of FBS compared to hPL. The differences between FBS and hPL were most pronounced at 10% supplementation, as FBS resulted in far fewer aggregates, which were larger and denser. hPL supplemented aggregates were more uniform, smaller and greater in number. A similar trend was observed at lower concentrations. These results suggest that hPL might support more physiologically relevant culture conditions, as denser clusters in FBS supplementation may promote the acquisition of culture-induced mutations and provide a competitive advantage for these clones.

While both Švajger (2017) and Jabbarpour et al. (2023) explored the effects of FBS and hPL on cell morphology, their findings highlight important nuances in supplement performance. Švajger (2017) reported no significant morphological differences between FBS and hPL in DC development, indicating comparable support for immune cell phenotypes. In contrast, Jabbarpour et al. (2023) observed distinct morphological disparities in CIK cells, with hPL producing more uniform and less dense aggregates than FBS. These findings imply that hPL may better preserve normal cell behavior and morphology, potentially reducing the risk of culture-induced anomalies. Together, the two studies that examine morphological differences between FBS and hPL supplementation point towards the possibility of morphological differences based on supplement concentration, as well as hematopoietic cell type.

### 3.3 Fold expansion

Fold expansion is the ratio between the final number of cells and the initial number of seeded cells. It is an important consideration for scaling HCC to clinically relevant volumes in applications where the total cell yield is the primary concern. The differentiation of PBMCs and CD34^+^ cells into desired cell types requires careful consideration of several key factors, including basal media, cytokine regime, additives, and stimulants. Table 1 summarizes these factors across the included studies. However, only three out of the five studies compared fold expansion in FBS and hPL supplementation. Two studies compared the expansion of lymphoid cells from PBMCs and one study compared fold expansion of myeloid cells from UCB-derived CD34^+^ cells.

**TABLE 1:** Cytokines, basal media, and additives used in included studies for the expansion of hematopoietic cell culture.

Cell type (lineage)	Basal media	Cytokine regime	Additives	Fold expansion	References
Dendritic cells (myeloid)	RPMI 1640	IL-4 (1,000 IU/mL) and GM-CSF (800 IU/mL)	Gentamicin (50 mg/mL) and GlutaMAX	Not applicable	Švajger (2017)
T-cells (lymphoid)	AIM-V	IL-2 (100 IU/mL), IL-7 (5 ng/mL), and/or IL-15 (5 ng/mL)	L-glutamine, streptomycin sulfate (50 μg/mL) and gentamicin sulfate (10 μg/mL)	↑ in FBS ↓ in hPL	Canestrari et al. (2019)
Megakaryocytes (myeloid)	IMDM	IL-3 (5 ng/mL), IL-6 (20 ng/mL), SCF (50 ng/mL), TPO (20 ng/mL)	Not applicable	↓ in FBS ↑ in hPL	Yaghoubi et al. (2021)
Erythrocytes (myeloid)	IMDM	IL- 3 (5 ng/mL), SCF (100 ng/mL), EPO (3 IU/mL)	L-glutamine (4 mmol/L), Iron-saturated human transferrin (220 μg/mL), ferrous sulfate (10 μg/mL), ferric nitrate (100 ng/mL), and insulin (20 μg/mL)	Not applicable	Zamani et al. (2021)
T-cells (lymphoid)	RPMI 1640	IL-2 (300 IU/mL), IFN- γ (1,000 IU/mL)	Penicillin/streptomycin (100 IU/mL) and anti-CD3 antibody (50 ng/mL)	↓ in FBS ↑ in hPL	Jabbarpour et al. (2023)

Abbreviations: CD-cluster of differentiation; EPO- erythropoietin; FBS- fetal bovine serum; GM-CSF- Granulocyte macrophage colony-stimulating factor; hPL-human platelet lysate; IL- interleukin; IMDM- Iscove’s Modified; LPS- lipopolysaccharide.

In myeloid cells, Yaghoubi et al. (2021) reported a small increase in the fold expansion at 10% supplementation using in-house hPL compared to FBS in CD34^+^ cells; however, this difference was not significant. Jabbarpour et al. (2023) reported a higher fold expansion of T-cells using in-house hPL compared to FBS across all three concentrations that were tested (2.5, 5, and 10%). Meanwhile, at 5% supplementation, commercially produced hPL decreased fold expansion compared to FBS (Canestrari et al., 2019). While there is limited data to enable meaningful comparison, the methods implemented to produce commercial hPL compared to in-house drastically differ and may account for the variability in the results obtained across studies.

Although only one study reporting fold expansion also performed biochemical profiling of hPL, it is reasonable to assume that growth factor concentrations can vary depending on the method of hPL production. Canestrari et al. (2019) found relatively low levels of IGF-1 (∼100 pg/mL) and TGF-β1 (150–200 pg/mL) across ten batches of commercially produced hPL. In contrast, Zamani et al. (2021) reported substantially higher concentrations of IGF-1 (111.1 ± 53.37 ng/mL) and TGF-β (27.18 ± 13.19 ng/mL) across five batches of in-house hPL, although they did not measure fold expansion in monoculture. It is worth noting that the values reported in in-house hPL are in range to reported levels of IGF-1 and TGF-β in FBS, which are 46 ± 1.6 ng/mL and 55 ± 0.6 ng/mL, respectively (Mohamed et al., 2020). ELISA was used to measure growth factor concentrations in all of these studies. Additionally, Canestrari et al. (2019) reported that commercial hPL was abundant in proteins involved in eliciting inflammatory and immune responses, which may suppress cell proliferation and contribute to lower fold expansion. The use of cryopreserved rather than freshly isolated PBMCs may further affect these results. Therefore, a comprehensive evaluation of hPL and FBS efficacy must consider production methods, growth factor profiles, and the source of PBMCs.

### 3.4 Cell viability

Cell viability, a measure of the proportion of live and healthy cells in a population, is an important indicator of cell culture performance. To compare the cell viability in hPL and FBS, annexin-V and 7-Aminoactinomycin D as well as trypan blue staining methods were used in the included studies.

Using double negative staining for annexin-V and 7-Aminoactinomycin D, Švajger (2017) measured cell viability at the end of the differentiation period (Day 6) of DCs that were cultured from PBMCs. Double negative staining is advantageous over other methods as it is accurate and sensitive, allowing the allowing distinction between live, early, late apoptotic/necrotic cells. Annexin-V is known to bind to phosphatidylserine in apoptotic cells as 7-Aminoactinomycin D stains the DNA in dead/late apoptotic cells, which have permeabilized membranes. Using flow cytometry, viability can be evaluated with more sensitivity and accuracy compared to other methods. The results demonstrated a 5% lower cell viability in hPL compared to FBS, but the results were not significant and showed more than 70% viability in both conditions.

Other staining methods reported higher viability in hPL and FBS supplemented cells. Trypan blue exclusion assay is a semi-quantitative method commonly used to obtain rough estimates of live versus dead cells in culture. Using Trypan blue exclusion assay, the viability of T-cells after differentiation in the presence of IL-2 from cryopreserved PBMCs (Day 14) was preserved (>90%) in both hPL and FBS, with no significant difference between the two conditions (Canestrari et al., 2019). Jabbarpour et al. (2023) reported a similar range, although it is unclear what method was used to obtain these results. Nevertheless, significantly higher viability was reported in hPL than FBS across three concentrations (2.5, 5, and 10%) at all time points. The viability in FBS was concentration-dependent, as lower viability was observed at concentration <10%, the least being at 2.5%. Meanwhile, viability remained >90% across all concentrations of hPL.

There is a lot of variability in the results regarding cell viability between the two supplementation groups, which may be attributed to hPL production method as well as method used to evaluate viability. In-house hPL presented the most variability ranging from 70% to >90%. However, similar results observed in FBS cultures, indicating that assay type may account for reported variability across studies. These results underscore the potential to replace hPL as an effective alternative for maintaining healthy cell populations in HCC but highlight the requirement for better reporting standards across cell culture experiments.

### 3.5 Cell identity

**FIGURE 2:**
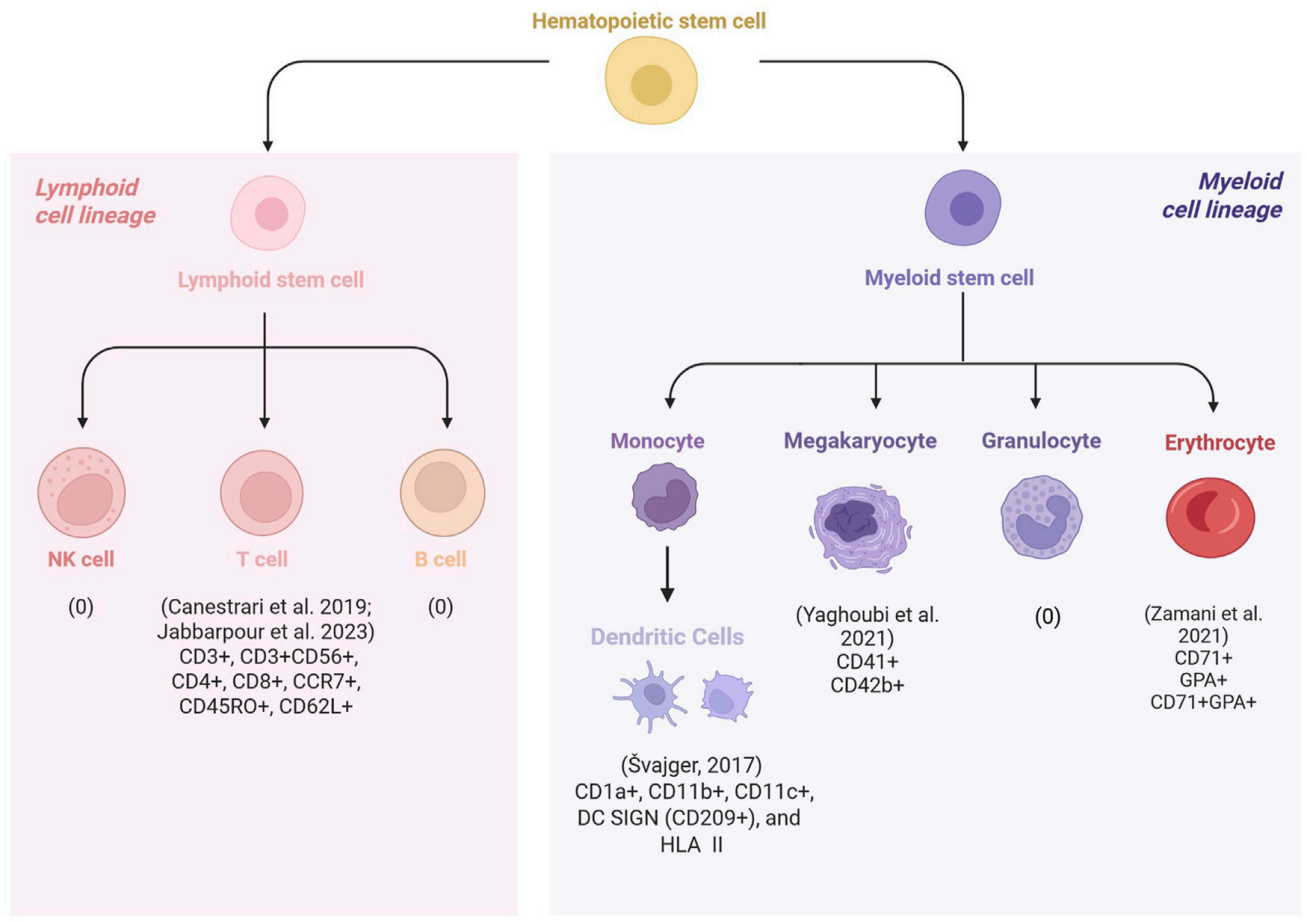
Schematic overview of the hematopoietic cell types used to evaluate human platelet lysate (hPL) as an alternative to fetal bovine serum (FBS). Across studies, cell identity was assessed by measuring the expression levels or phenotypic prevalence of cells, using archetypal surface markers. None of the included studies investigated the use of hPL in the culture of natural killer (NK) cells or granulocytes. Jabbarpour et al. (2023) examined cytokine-induced killer (CIK) cells, a hybrid population with both T-cell and NK-like features and found that hPL increased the proportion of cells expressing characteristic CIK markers (CD3^+^/CD56^+^). Canestrari et al. (2019) reported that except for naïve cells (CCR7+/CD45RO-), a higher proportion of T cell subsets were obtained in hPL-supplemented cultures compared to FBS. Yaghoubi et al. (2021) and Zamani et al. (2021) observed increased expression of megakaryocyte and erythroid markers, respectively, in hPL compared to FBS supplementation. Švajger (2017) assessed immunophenotypic changes in dendritic cells (DC) by measuring marker expression levels. Compared to FBS, hPL supported DC maturation from immature to mature states, with higher ability to promote the tolerogenicity in culture.

One of the challenges in HCC is the spontaneous commitment to differentiated phenotypes, which makes it difficult to obtain relevant volumes of desired cell types. Hematopoietic cells can be differentiated by their unique cell surface protein profiles. Using flow cytometry, phenotypic prevalence and expression level of markers can provide information on the functional and differentiation status of cultured cells. Best practices include reporting both parameters; however, in studies where yield was a priority, only phenotypic prevalence was reported. On the other hand, studies where immunophenotypic changes during differentiation were important, expression levels of markers were presented. Table 2 summarizes the findings of the studies that reported phenotypic prevalence across hPL and FBS groups. Figure 2 illustrates the various cell types examined across the included studies. Although cell morphology, fold expansion, and viability results varied drastically across studies, hPL supplementation had a positive effect on maintaining cell identity across all the cell types that were evaluated in the included studies.

**TABLE 2:** Effect of Supplement on the Expansion of Different Hematopoietic Cell Subtypes. The table shows the final percentage of expanded cells for various cell subtypes cultured with human platelet lysate or fetal bovine serum.

Subtype	Marker	Supplement conc	Final percentage in expanded cells		References
			Human platelet lysate	Fetal bovine serum	
T	CD3^+^ CD56^−^	5%	97.3%	91.9%	Canestrari et al. (2019)
T_Central Memory_	CD4+/CCR7+/CD45RO+	5%	42.3%	13.7%	
	CD8+/CCR7+/CD45RO+	5%	36.6%	17.2%	
T_Naive_	CD4+/CCR7+/CD45RO-	5%	No difference observed		
	CD8+/CCR7+/CD45RO-	5%	No difference observed		
Homing	CD62L+	5%	84.3%	73.7%	
Megakaryocyte	CD41^+^	10%	45.8%	37.8%	Yaghoubi et al. (2021)
	CD42b+	10%	13.2%	10.5%	
	CD41+/CD42b+	10%	12.1%	9.5%	
Erythrocytes	CD71^+^	10%	94.0%	90.0%	Zamani et al. (2021)
	GPA+	10%	66.2%	61.3%	
	CD71+/GPA+	10%	65.0%	60.3%	
	CD71-/GPA+	10%	1.2%	1.0%	
Cytokine-induced killer	CD3^+^ CD56^+^	2.5%	31.2%	21%	Jabbarpour et al. (2023)
		5%	42.8%	28.8%	
		10%	52.6%	54.7%	

Abbreviations: CCR-chemokine receptor; CD-cluster of differentiation.

Švajger (2017) demonstrated that by Day 6, hPL supplementation effectively supported the generation of iDCs with high expression of characteristic markers, evaluated by flow cytometry. Expression of monocyte-associated markers (low CD14 and CD16; high CD1a and DC-SIGN) was comparable between FBS- and hPL-supplemented cultures, although CD1a expression was notably reduced with hPL. The DC-associated markers CD11b and CD11c were expressed at higher levels in FBS conditions. Evaluation of HLA molecules revealed similar HLA-I expression levels between groups, while HLA-II expressions were elevated in hPL cultures. Following activation with lipopolysaccharide (20 ng/mL), the expression of co-stimulatory molecules CD40 and CD83 was lower in hPL-supplemented cells, whereas CD80 and CD86 levels remained comparable. Notably, the lymph node-homing receptor CCR7 and HLA-II, indicative of DC maturation, were slightly higher in hPL conditions. Furthermore, upon treatment with 1,24-dihydroxyvitamin D_3_, ILT-3 expression, associated with tolerogenic potential, increased by two-fold in hPL cultures compared to FBS. Collectively, these findings suggest that hPL is a suitable alternative to FBS for DC differentiation and may offer advantages in promoting a TolDC phenotype.

Canestrari et al. (2019) reported that compared to FBS supplementation, hPL has a capacity to produce T cells of less differentiated subtypes. In this study, T-cell maturation was measured after CD3/CD28 stimulation by immunostaining for CD62L or a combination of CCR7 and CD45RO to assess CD4^+^ and CD8^+^ T-cell subtypes. As shown in Table 2, hPL consistently showed a significantly increased proportion of cells expressing CCR7 and CD45RO, indicative of the central memory phenotype (T_CM_). Additionally, an increased percentage of CD62L^+^ cells was observed, further supporting the enrichment of both naïve and T_CM_ cells in hPL cultures. These findings suggest that hPL not only supports T cell expansion but also promotes a more desirable, less terminally differentiated phenotype, which may be advantageous in adoptive immunotherapy applications.

Yaghoubi et al. (2021) evaluated megakaryocyte lineage markers from UCB-derived CD34^+^ cells using flow cytometry on Day 8. Significantly higher proportion of cells expressed CD41 marker in hPL compared to FBS supplementation. A slight increase in the proportion of cells expressing CD42b in hPL was also observed; however, the difference was not significant. CD41 and CD42b are megakaryocyte markers, and the increase observed in hPL compared to FBS supplementation, potentially suggest enhancement of proplatelet formation. Similarly, Zamani et al. (2021) reported an increase in the percentage of cells expressing early to intermediate erythroid progenitor markers, CD71 and glycophorin A, in hPL compared to FBS on Day 7. These findings suggest that hPL not only supports lineage-specific differentiation but also promotes the maintenance of more desirable, less terminally differentiated progenitor cell types compared to FBS, which may enhance both the efficiency and functional potential of HCC.

Jabbarpour et al. (2023) evaluated cell identity of CIK cells across three supplement concentrations (2.5, 5, and 10%). Archetypal cell marker of CIK cells reflects their hybrid T/natural killer-like phenotype. At lower concentrations, the proportion of cells expressing CIK markers, CD3 and CD56, were lower than at 10% in both cultures. However, at 5% supplementation, the proportion of cells expressing these markers were double that of FBS (Table 2). T-cell marker expression (CD3^+^CD56^−^) was comparable between both cultures and across all three concentrations (>95%). Canestrari et al. (2019) reported similar results as no significant difference was observed in the proportion of T-cells (CD3^+^CD56^−^), although the percentage of cells was slightly higher in hPL than FBS. These results suggest that hPL is an effective alternative to FBS in obtaining differentiated phenotypes of lymphoid lineages.

Overall, the studies demonstrated that hPL is a viable and, in many cases, advantageous alternative to FBS in maintaining the identity and desirable phenotypes of hematopoietic-derived cells. hPL supplementation supported the expression of lineage-specific surface markers of DC, T cells, megakaryocytes, erythroid progenitors, and CIK cells. Moreover, hPL allowed the retention of less terminally differentiated states, including TolDCs, T cells, and early-stage erythroid and megakaryocytic progenitors. Together, these findings highlight hPL’s potential to enhance both the functional quality and translational relevance of HCC for clinical applications.

### 3.6 Functional assays

#### 3.6.1 Cytotoxic assay

Cell-mediated cytotoxicity assays are a good measure of functionality of cultured immune cells (effector) to kill cancer or infected cells (target). Jabbarpour et al. (2023) evaluated the cytotoxic effect of 5% and 10% hPL supplemented CIK cells on Day 15. Two hematopoietic cancer cell lines, K-562 and Raji were used as targets, which were labeled using 5 μg/mL Carboxyfluorescein succinimidyl ester (CFSE) dye in 5% FBS in phosphate buffer saline (PBS). The cells were then incubated in a water bath (at 37°C for 10 min) and the reaction was neutralized by adding RPMI and 10% FBS. Target cells were then washed with PBS and mixed with CIK cells to make three effector-to-target ratios (40:1, 20:1, and 10:1). These co-cultures were performed in RPMI containing 10% FBS and allowed to grow overnight at 37°C and 5% CO_2_. The next day, cell death was measured using propidium iodide staining, which penetrates dead or damaged cells and binds to the DNA. Cells were then incubated with a propidium iodide working solution (including RNase A and Triton X-100) for 30 min at 4°C in the dark. Using flow cytometry, the results showed that there was no significant difference between 5 or 10% hPL supplemented cells and both groups showed high percentages of dead cells across all three tested ratios. These results suggest that a wide range of hPL concentrations can be used to culture CIK cells without affecting their cytotoxic effect on hematopoietic cancer cells. However, the results do not adequately shed light on the effectiveness of using hPL as an FBS alternative because no FBS controls were maintained. Further, it should not be assumed that switching between hPL to FBS supplementation would not have profound impact on cells metabolism (Bieback, 2013). Hence, these results are confounded by the fact that CSFE and propidium iodide staining do not discriminate between the mechanism of cell death or killing. Future studies can increase the reliability of such data by maintaining FBS controls as well as avoiding sudden switching between hPL to FBS during experimentation.

#### 3.6.2 Transduction efficiency

Transduction efficiency describes how well cells can take up genetic material. High transduction efficiency is an advantage for gene therapy and cell engineering applications where robust gene expression is required. Culture conditions can have a significant effect on transduction efficiency. Canestrari et al. (2019) examined the effect of supplementation on transduction efficiency of primary T cells. The effect of FBS and hPL on transduction efficiency were compared using a green fluorescent protein (GFP) reporter gene, whose expression was driven by three different promoters (CMV-GFP, PGK-GFP, and EF1α-GFP). To assess the differences between culture conditions, the multiplicity of infection (MOI) was maintained at <1; however, it was not standardized across vectors due to promoter differences. Nevertheless, viral entry and stable gene integration were measured using flow cytometry on Days 2 and 7, respectively. Data was reported based on two experimental repeats.

In the first set of experiments, the transduction efficiency, which was expressed as the percentage of cells expressing GFP, varied based on donor T-cells with no major differences between Day 2 and 7. A second set of transduction efficiency experiments were performed using different donor cells and fresh batches of hPL, which showed an increase in the percentage of cells expressing GFP. Hence, the study concluded that hPL might improve transduction efficiency depending on donor cells and freshness of hPL batches. Across both sets of experiments, the mean fluorescence intensity measured was higher in hPL-supplemented cells than in FBS. In the second set of experiments, the mean fluorescence intensity was ∼20-fold higher than in FBS on Day 7. The increase in expression was independent of promotor used, although the increase in EF1α-GFP, which is commonly used in clinical chimeric antigen receptor T (CAR-T)- cell constructs, was modest (<2-fold). Therefore, this study demonstrates that hPL may not enhance transduction efficiency but increases transgene expression per cell compared to FBS.

#### 3.6.3 Gene expression

Zamani et al. (2021) evaluated the effects of hPL and FBS supplementation on erythrocyte differentiation from UCB-derived CD34^+^ cells. Cells were cultured in IMDM supplemented with 10% FBS or hPL, with stabilized glutamine, transferrin, iron salts, and insulin. Results showed that the expression levels of key erythroid genes, including GATA-1, NFE-2, and the globin genes γ and β, were significantly higher in hPL-supplemented erythroid cells than in those cultured in FBS. This enhancement in erythroid differentiation and gene expression suggests that hPL may offer superior support for erythropoiesis, making it a promising supplement for applications requiring robust production.

Yaghoubi et al. (2021) compared the effect of hPL and FBS on megakaryocyte progenitor gene expression profile. UCB-derived CD34^+^ cells were cultured in IMDM and 10% FBS or hPL for 7 days. Compared to FBS, hPL cultures were found to express elevated levels of GATA-1, GATA-2, FLI-1, NFE-2, and RUNX1. These results suggest that hPL is more effective than FBS at promoting the expression of key genes required for megakaryocyte differentiation, supporting its potential use as an FBS alternative in hematopoietic cell culture.

#### 3.6.4 Endocytosis

The endocytic activity of DCs is fundamental to their function as antigen presenting cells. By capturing, processing and then presenting antigens to the killer T cells, they are a vital line of defense in the innate immune response. Švajger (2017) evaluated the effect of supplementation on the endocytic activity of both iDCs and mDCs, using flow cytometry. However, no differences were observed in the ability of iDCs to endocytose FITC-Dextran in both FBS and hPL supplementation. While this ability was high in case of iDCS, the mDCs showed very low endocytotic activity in both hPL and FBS supplementation (Švajger, 2017). Thus, it can be inferred that hPL does not interfere with the functional capacity of DCs, preserving its ability to perform as APCs.

## 4 Recommendations on enhancing hPL production reporting for HCC applications

**FIGURE 3:**
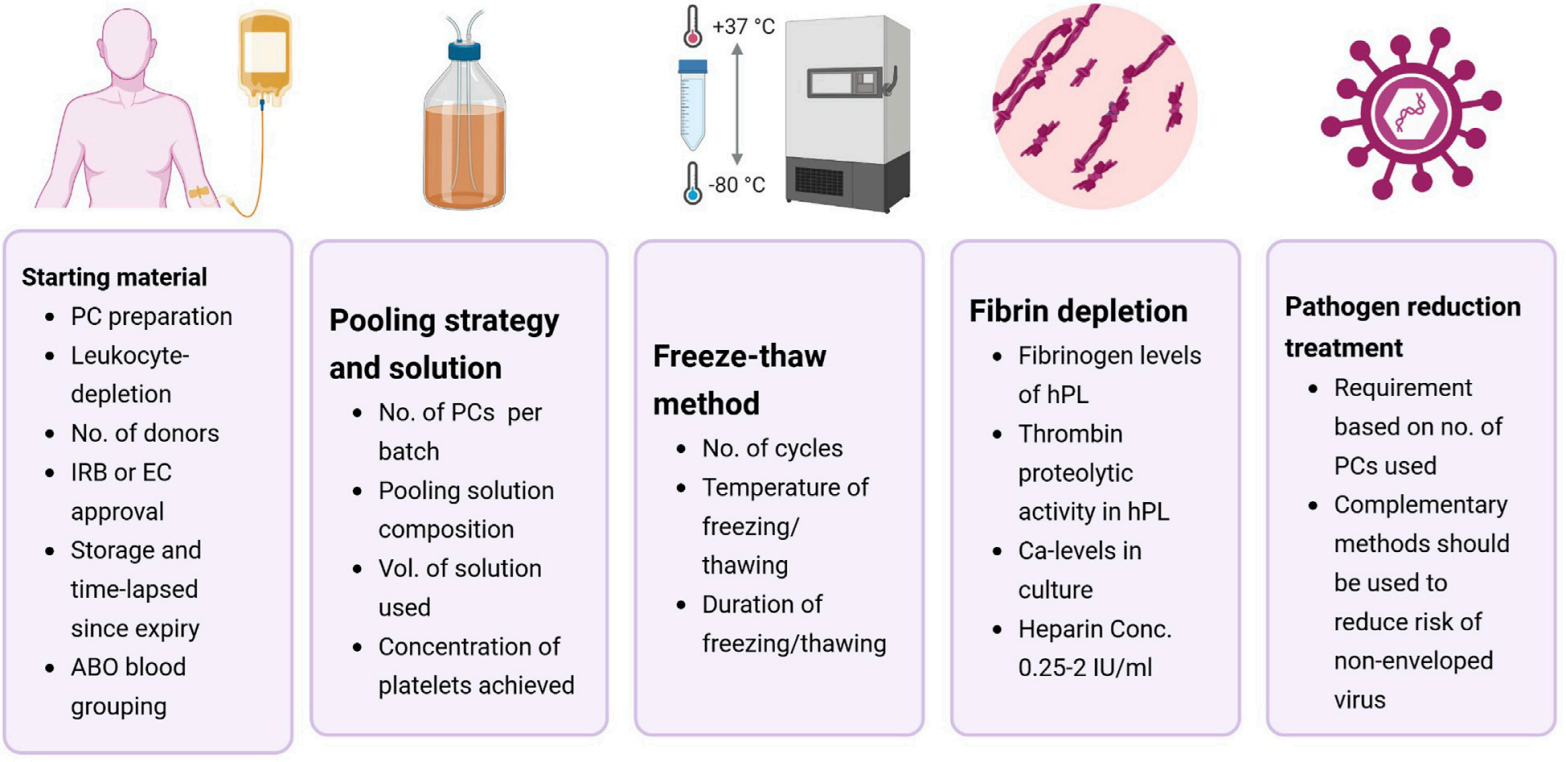
Recommendations for standardizing human platelet lysate (hPL) production parameters for hematopoietic cell culture. Most of the included studies lacked sufficient methodological detail to allow reproducibility by third parties. To improve standardization, future studies should clearly report key aspects of hPL production, including details on the starting material, pooling strategy and solution, lysis method, fibrin depletion, and pathogen reduction treatments. Abbreviations: EC-ethical committee; IRB- institutional review board; PC-platelet concentrates.

hPL is the final product obtained from the mechanical lysis of platelet concentrates (PC; Chisini et al., 2017). PCs are routinely prepared using platelet-rich plasma (PRP), buffy-coat (BC), or apheresis. A more systematic analysis of the effectiveness of hPL as an FBS alternative was not possible as details regarding hPL production were notably scarce and inconsistent across the included studies. The efficacy of hPL may be affected by production parameters and there is a need to enhance reporting standards (Chou and Burnouf, 2016; Bieback et al., 2019; Henschler et al., 2019; Palombella et al., 2022). Table 3 summarizes the different variables of hPL production across the included studies. Figure 3 illustrates the recommendations on enhancing the reporting standards of hPL production methodology for HCC.

**TABLE 3:** Overview of the parameters of human platelet lysate (hPL) production methods implemented across the included studies.

References	Starting material	No. of donors	Pooling		Lysis	Leukocyte depletion	Fibrinogen depletion	Pathogen reduction treatment
			Strategy	Solution				
Švajger (2017)	Apheresis	3	-	-	1 F/T at −80°C and +2°C–8°C	Yes	No	No
Canestrari et al. (2019)	Apheresis	100	-	-	E-beam irradiation	Yes	No	Yes
Yaghoubi et al. (2021)	Expired PRP	-	10 PC	-	3 F/T at −80°C and +37°C	Yes	No	No
Zamani et al. (2021)	Expired PRP	-	25 PC	-	3 F/T at −80°C and +37°C	Yes	No	No
Jabbarpour et al. (2023)	PRP	-	-	-	3 F/T at −80°C and +37°C	Yes	Yes	No

Abbreviations: BC: buffy coat; F/T: Freeze-thaw; PC: platelet concentrate; PRP: Platelet-rich plasma.

### 4.1 Starting material

#### 4.1.1 Ethical and legal considerations of using PCs

Blood banks routinely prepare PCs to treat patients with severe thrombocytopenia. However, there are ethical concerns about diverting life-saving blood products away from patients to meet the growing demand for hPL. To avoid such a scenario, it is recommended to use expired PCs for hPL production (Weber et al., 2022). PCs have a short shelf-life (<5 days) and often expire, causing a substantial burden on healthcare systems. Hence, hPL has garnered attention as it offers an attractive opportunity to repurpose expired PCs into a viable human cell culture supplement (Burnouf et al., 2014). Some studies also use fresh PCs to prepare hPL. In such cases, PCs are prepared from a small number of voluntary blood donations after obtaining informed consent, as well as approvals from an institutional review board or ethical committee. Moreover, donor privacy and confidentiality are ensured according to local regulations and guidelines (Jacobs et al., 2019). Therefore, the concerns regarding hPL production are safeguarded by ethical and legal considerations of human biological sample handling. Only three of the five included studies clearly reported compliance with these considerations, highlighting the scope of ensuring transparency in such studies.

#### 4.1.2 PC preparation method

Three studies used PRP-PC, while one study used the BC-PC, and another study used apheresis. All the included studies performed leukocyte depletion by filtration. In MSCs, PRP, BC, and apheresis had no effect on cell proliferation and had similar mean doubling times (Palombella et al., 2022). However, a higher variability existed across studies that used PRP-PC and BC-PC. Compared to apheresis, PRP-PC and BC-PC have a higher risk of leukocyte contamination. Cañas-Arboleda et al. (2020) demonstrated that by adjusting platelet concentrations and reducing leukocyte levels, variability was less across hPL batches. Specifically, they observed that adjusted pools that had leukocyte levels below 600/μL, which contributed to a more stable pH and reduced immunogenicity, creating an optimal environment for MSC proliferation. Future studies would benefit from monitoring the effects of leukocyte levels on batch variability.

#### 4.1.3 ABO grouping

None of the included studies provided any information on the blood group of PCs. While ABO compatibility may not be an important consideration for some cell types, such as T cells, ignoring it could be problematic for cells that express ABO antigens (Burnouf et al., 2016; Oeller et al., 2021). Nevertheless, compared to FBS, hPL enhanced the production of megakaryocyte progenitors and functional erythroid cells from purified UCB-derived CD34^+^ cells (Yaghoubi et al., 2021; Zamani et al., 2021). While hPL supports HCC, these gaps in standardization and reporting practices regarding the starting material need to be clarified.

#### 4.1.4 Storage and time-lapsed since expiry

While some studies indicate the use of expired platelets obtained from national blood banks (Yaghoubi et al., 2021; Zamani et al., 2021), others prepare PCs from fresh whole blood samples. Shanbhag et al. (2020) demonstrated that hPL from expired PCs stored for up to 4 months were able to efficiently support the proliferation and differentiation of MSCs, after which major cytokines reduced and affected cell culture performance. In a study comparing shorter storage times, the pH, glucose content, and albumin content of hPL were most affected by longer storage times (>5 days) of expired PCs; however, MSC proliferation rate was not affected (Mentari et al., 2022). Except for one study, where PCs were frozen at −80°C within 2 days of expiry, the remaining studies did not report any details regarding storage and time-lapsed since expiry. Therefore, these studies not only emphasize the need to better define starting material for hPL in HCC applications but also are an opportunity to enhance reproducibility in vitro studies.

### 4.2 Pooling strategy and solution

Pooling strategies and solutions also vary widely across studies, contributing to differences in hPL quality and batch-to-batch variability. Zamani et al. (2021) reported high batch-to-batch variability in the levels of growth factors measured across five batches, which pooled five PCs each. Canestrari et al. (2019) suggest that using at least 100 donations per batch may overcome biological variation arising from donors. Viau et al. (2019) reported that across 14 batches, pooling 200 donations in additive solution, containing 25%–40% residual plasma, reduced the coefficient of variance (CV) <9%. Whereas using only five donations had a greater CV of 18%–30%. Conversely, Fazzina et al. (2015) demonstrated that pooling 12 PC donations in ∼600 mL of InterSol solution (Fenwall Inc.) and 20%–30% human plasma showed no relevant variation in the growth factor concentration across 20 batches. More recently, Bianchetti et al. (2021) recommended pooling 25 PC donations in cryoprecipitated plasma reduced CV <9%. However, Zamani et al. (2021) reported high batch-to-batch variability using 25 donations. Therefore, a systematic study that evaluates the effect of pooling strategies and solutions is needed to standardize hPL production methods.

### 4.3 Freeze-thaw cycles and platelet lysis

Most studies used a simple and cost-effective method of repeated freeze-thaw cycles to break down platelets for hPL preparation. Specifically, three out of five HCC studies used a three-cycle protocol at −80°C for freezing and +37°C for thawing. One study found success with just a single freeze-thaw cycle at −80°C and +2°C–8°C (Švajger, 2017). Additionally, Canestrari et al. (2019) used electron beam (E-beam) irradiation to lyse the cells. No other study implemented pathogen reduction treatments. There was little evidence to suggest that one platelet lysis method may provide a better hPL biochemical profile than another. Instead, the choice of methods implemented by different groups was based on cost-effectiveness and convenience on handling volumes. Bianchetti et al. (2021) found that lower cycle counts increased variability in growth factor release, while four cycles achieved optimal stability and growth factor consistency. Similarly, across MSC studies, doubling time was lower in hPL produced from at least three cycles (Palombella et al., 2022). Therefore, standardizing protocols to perform three to four freeze-thaw cycles for platelet lysis could reduce variability and enhance hPL’s effectiveness in HCC.

### 4.4 Fibrin depletion

Plasma contains a variety of clotting factors, including fibrinogen and prothrombin. Prothrombin is converted to thrombin, which produces fibrin from fibrinogen. Thus, fibrin depletion or control is recommended when using hPL to prevent the gelation of culture media. Hemeda et al. (2013) reported that the addition of heparin was critical to maintaining low-glucose, DMEM in a liquid state for MSC culture. The hPL used in this study was made from PCs, which were prepared by suspending 2.0–4.2 × 10^11^ platelets in 200 mL plasma containing acid-citrate-dextrose (ACD). PCs were subjected to two freeze-thaw cycles. However, heparin can reduce cell viability, alter cell behavior, and significantly affect miRNA expression (Hemeda et al., 2014; Ling et al., 2016; Oeller et al., 2024).

Heparin is commonly obtained from porcine or bovine mucosal tissue, which can complicate clinical applications and introduce variability in hPL-supplemented cell culture experiments. To develop a completely xeno-free culture, Mojica-Henshaw et al. (2013) investigated the feasibility of converting hPL-plasma to hPL-serum for MSC culture. hPL-plasma was obtained by subjecting PC (1.0 × 10^9^ platelets/mL) to a freeze-thaw cycle. For hPL-serum, CaCl_2_ was added to PC and allowed to clot overnight. The clot was removed, and the supernatant was collected. In MSCs cultured in α-MEM, both hPL-plasma and serum resulted in similar immunophenotype profile and tri-lineage differentiation; however, hPL-plasma resulted in higher cell yields compared to hPL-serum. Thus, the need for heparin may depend on the hPL production method, which could leave the fibrinogen and coagulation factors intact.

Typically, the fibrinogen levels reported in hPL produced using freeze-thaw cycles, sonication, and solvent-detergent chemical treatment ranged from 1.5 to 3.0 g/L, which requires the addition of 0.6 to 2.0 IU/mL of heparin in the cell culture media (Chou and Burnouf, 2016). Meanwhile, methods including serum conversion and removal of clotting factors through the addition of CaCl_2_ may not require heparin addition. In a study that directly compared the two methods, fibrinogen was present in freeze-thawed hPL, but no thrombin proteolytic activity was detected (Delila et al., 2021). On the other hand, fibrinogen was <0.4 mg/mL in serum-converted hPL (using 23 mM of CaCl_2_) and high thrombin proteolytic activity was observed. The activity significantly reduced in heated serum-converted hPL, which may suggest that heat might affect thrombin activity.

A recent systematic review evaluating the effectiveness of hPL to replace FBS in MSC, reported that four out of the 29 included studies did not add heparin during hPL production nor to cell culture (Palombella et al., 2022). The absence of heparin did not affect the doubling time. Mohamed et al. (2020) reported that cultures with Ca content <0.25 mM could not induce clot and did not require heparin. In this study, no gelation was observed in RPMI-1640 (0.4 mM of Ca) whereas heparin at a minimum concentration of 0.25 IU/mL was required for DMEM (1.5 mM of Ca) cultures. Two of the five studies that were included in this review used RPMI-1640 for HCC and one study added 2 IU/mL (Jabbarpour et al., 2023). Meanwhile two other studies used IMDM (1.5 mM of Ca) and did not add heparin or implement any other measures. AIM-V was used as a basal media in one study, and the composition of this media is not publicly available.

Given the variability in the application of fibrinogen depletion across studies, greater transparency and consistency across studies could be achieved by reporting key parameters that enable comparison. These should include the fibrinogen concentration and thrombin proteolytic activity of the hPL, as well as the final Ca concentration in the culture medium. Standardized reporting of these factors would help clarify their impact on experimental results and support reproducibility across research settings.

### 4.5 Pathogen reduction treatment

Although most of the hPL production process is performed in a closed loop, and the starting material undergoes sterility checks according to national and international blood bank requirements, PCs are susceptible to pathogen contamination. While risks are lower in hPL produced using small pooling strategies, contamination from a single donation can have disastrous effects in larger pooling strategies (>16 donations). However, the selection of pathogen reduction method and frequency may affect hPL performance in cell culture (Bieback et al., 2019). Studies confirm that the composition of hPL is affected by pathogen reduction treatment, but cell culture performance remains unaffected (Viau et al., 2017; González et al., 2022). There is some evidence of failure especially regarding non-enveloped viruses and the application of complementary reduction methods is recommended for large pooling strategies (Blümel et al., 2020). Therefore, careful selection and application of pathogen reduction treatments can ensure the safety and effectiveness of repurposing large volumes of expired PCs and enable further studies to better compare hPL and FBS in HCC.

## 5 Discussion

This review highlights the need for more robust hPL production protocols and increased consistency in reporting standards that will enable a systematic comparison between FBS and hPL in HCC. Due to cost and availability, hPL is often prepared using PRP-PCs, where the variability in ABO grouping, storage times, and conditions of expired PCs need to be clarified. Most studies employ three freeze-thaw cycles at −80°C and +37°C to lyse platelets; however, pooling strategies and solutions are rarely described with adequate detail to enable reproduction by third parties. Fibrin depletion, generally achieved by heparin addition, is frequently omitted, though its necessity in hPL-supplemented HCC should be clarified based on Ca content, thrombin proteolytic activity, and basal media used. Similarly, the need for pathogen reduction treatment needs to be clarified based on the pooling strategy, storage times of expired PCs, and intention for clinical applications. Overall, consistent protocols across these factors are necessary to optimize hPL’s efficacy and reproducibility in HCC applications.

Across all cell types, the proportion of cells and expression levels of archetypal surface proteins increased in hPL compared to FBS. Surface proteins participate in several biological processes including metabolism, cell-to-cell communication, and immune responses. While the association between upregulated surface proteins and cell culture stability is yet to be investigated in HCC, it has been previously observed in MSC (Reis et al., 2018). Of the 99 surface proteins found in hPL and FBS-supplemented MSCs, 48 were enriched in the former, leading to increased homogeneity in the culture. Additionally, compared to hPL, FBS significantly increased the levels of IL-8 production in DCs by 30% (Švajger, 2017). This cytokine is associated with pathological conditions as it participates in pro-tumorigenic responses (Fousek et al., 2021). Liu et al. (2023) demonstrated that across eight brands of FBS, the background IL-8 secretion in epithelial cells (HCT-8) varied. Small molecules (<3 kD) in FBS promoted IL-8 secretion by activating the pERK pathway in epithelial cells. As most hematopoietic cell types are sensitive to IL-8, evaluating surface markers and cytokine expression in response to FBS may provide insight into the sources of HCC instability.

HCC expansion may introduce genetic mutations during cell division and telomere shortening. Concerns persist regarding chromosomal abnormalities and the preferential expansion of preleukemic mutations linked to clonal hematopoiesis (Meaker and Wilkinson, 2024). Regarding hPL supplementation, Trojahn Kølle et al. (2013) reported that no chromosomal aberrations were detected in all cultured MSCs. Fazzina et al. (2015) demonstrated that doubling times were increased in hPL compared to FBS across cell lines and were statistically significant for leukemic cell lines, JURKAT and KG-1a. In these cell lines, increased proportions of S- and G0/G1-phase cells were observed in FBS and hPL supplementation, respectively. Similar results have been shown in other cancer cell lines comparing FBS and xeno-free supplements. Human hepatoma cells cultured in human serum stopped proliferating, assumed hepatocyte-like morphology, and replaced the Warburg-like metabolic profile typically observed in FBS-cultured cells (Steenbergen et al., 2018). These findings suggest the importance of studying cell penalties due to media and supplementation.

Driven by the potential of harnessing the immune system and developing cancer treatments, the interest in developing adoptive cell therapies has increased in recent years (Rohaan et al., 2018). These cell types are required in large quantities to be clinically relevant; thus, requiring high volumes of media and sera. While the cost of both supplements varies based on manufacturers, the price differences between FBS products are drastic based on quality assurance, sterility, and geographical origin (Gstraunthaler et al., 2013; Chelladurai et al., 2021). In MSCs, concentrations as low as 0.75% hPL showed equivalent performance to 10% FBS supplementation (Cowper et al., 2019). Two studies examined hPL performance to FBS at 5% supplementation, and this trend was not observed as the fold expansion in FBS was higher than that in hPL. Hence, the effective minimal concentration of hPL still needs to be determined for HCC applications.

More recent attention has focused on developing CAR-T cells, which not only require large amounts of raw materials but also need optimized transduction protocols. Compared to other systems, EEF1α lentiviral systems have been shown to have increased transgene expression in differentiated hematopoietic cells (Salmon et al., 2000). The EEF1α system showed higher transgene expression in hPL compared to FBS supplementation. The trend was observed regardless of the lentiviral system used; however, this was not the case in SFM. In SFM, EEF1α-based transgene expression was lower than in other systems (Sutton et al., 2016). Another desirable characteristic in CAR-T cells is the presence of a high proportion of central memory T cells, which was observed in hPL supplementation (Canestrari et al., 2019). Similar results were reported in CAR T-cells expanded in hPL, which demonstrated a less differentiated phenotype, superior proliferation, and enhanced anti-tumor effects (Chavez et al., 2019). These results suggest that hPL supplementation can yield a more robust expression of transgenes to ensure a long-term tumor-killing effect, with a low multiplicity of infections (<1).

Nevertheless, while hPL presents advantages over FBS, it is important to recognize that both are undefined biological materials with inherent variability and risks related to batch-to-batch differences and potential contamination. In the long-run, it is desirable that the field should transition toward the use of fully chemically defined media, particularly in the context of sensitive clinical applications such as CAR-T and other cell therapies. Additionally, during periods of global viral outbreaks, such as the COVID-19 pandemic, ensuring the safety and consistency of culture media becomes even more critical. Chemically defined media may provide the level of reproducibility and biosecurity required for future-proof and scalable cell therapy production. Oredsson et al. (2025) present a comprehensive protocol for the use of the Oredsson Universal Replacement (OUR) medium, a xeno-free, chemically defined, open-source medium designed for culturing a range of human normal and cancer cell lines. The protocol outlines procedures for thawing, culturing, and freezing cells in a reproducible and serum-free environment. Although the study does not directly compare OUR medium with FBS or hPL, it reports that the performance benchmarks for CD4^+^ T cells, Jurkat, and THP-1 cultured in OUR medium were consistent with existing literature.

In conclusion, this review highlights the need for the implementation of more robust hPL production protocols and enhanced reporting standards to facilitate a systematic comparison with FBS in HCC studies. Despite considerable methodological biases, hPL still offers a promising alternative to FBS in HCC. At 10% supplementation, hPL demonstrated superior culture stability, enhanced plasticity, and fold expansion compared to FBS. Therefore, hPL may offer a cost-effective solution to large-scale *ex vivo* expansion of HCC. Future studies should aim to implement robust methods and transparent reporting as discussed here, while also considering and supporting the long-term shift toward chemically defined media.

## Data Availability

The original contributions presented in the study are included in the article, further inquiries can be directed to the corresponding author.
